# Land Use and Land Cover Dynamics: Deriving Forces and Perceptions of Local Community in Derashe, Southern Ethiopia

**DOI:** 10.1155/2023/6905404

**Published:** 2023-12-26

**Authors:** Agegnehu Taye, Abiyot Anbacha, Firehiywet Girma

**Affiliations:** ^1^Hawassa University, College of Agriculture, Department of Rural Development and Agribusiness, Awasa, Ethiopia; ^2^Hawassa University, Wondo Genet College of Forestry and Natural Resource, Department of Geographic Information Science, Awasa, Ethiopia

## Abstract

Changes in land use and land cover (LULC) reached a point where we must reconcile. Thus, this study was conducted to assess the LULC changes, deriving forces, and the local community perceptions in Derashe, Southern Ethiopia. Landsat Series sensors' 4, 5, and 8 imagery was used to analyze the LULC over the year. Focus group discussions (FGDs) and key informant interviews (KIIs) were used to assess the deriving force and perceptions. Maximum likelihood of the supervised digital image classification technique was employed. Postclassification was used to detect and quantify changes. Deriving force and perception data were analyzed through descriptive statistics and narration. Five major LULC classes were identified. A major increase was observed in settlement and agricultural land by displacing forest and bush lands. However, the water body was fluctuated. According to the local community, the agricultural land expansion and growing population pressure are the major proximate and underlying deriving forces, respectively. Therefore, proper design for efficient use is necessary.

## 1. Background of the Study

Land is the most important natural resource and a fundamental factor of production on which economic, social, infrastructural, and other livelihood-related activities such as food production, shelter, infrastructure development, and natural resource extraction take place. However, land resources are becoming increasingly scarce around the world due to continued exploitation and mismanagement [1]. Land use/land cover changes have occurred at all times in the past, are currently underway, and are likely to continue in the future [1, 2]. Estimates suggest that global forest cover has declined by about 1.8 billion hectares over the past 5,000 years (a decline equivalent to nearly 50% of total forest cover today) [3], and global forest cover declined by 129 million hectares (3.1%) to nearly 4 billion hectares during 1990–2015 [4]. This increasing bio-diversity loss combined with high demand for land-based goods and services and the manner in which they are produced today are adversely impacting the health and future productivity of the planet [5]. And thus “It is time for a change in consciousness,” and it is a fact that agriculture and forestry can no longer be treated in isolation. Linking the two is imperative for socioeconomic development in the 21st century” [4]. The huge demand for commodities and services generated on land as well as how they are now produced are all having a detrimental effect on the planet's productivity now and in the future [5]. As a result, it is “time for a shift in consciousness,” and the separation of agriculture and forestry from other sectors of the economy is no longer appropriate. For socioeconomic development in the twenty-first century, linking the two is crucial [4].

The degradation and loss of forests and woodlands, the extinction of animal and plant species, the degradation of land, the rise in water scarcity, and the degradation of water quality all tend to go hand in hand with the use of natural resources in Africa, where 60% of the population lives in rural areas and 70% of the economically active population depends on agriculture (a natural source) [6]. With the exception of Guinea-Congo and Zambezia, the most significant changes in land cover occurred between 1975 and 2000, when agricultural land increased by 57% at the expense of forests and nonforested natural vegetation, which together made up 55% and 45% of the total area. This corresponds to a loss of about 5.2 ha (52,000 km^2^) of natural vegetation annually. Ethiopia, one of the most populated nations in Africa, is the most severely impacted [7], with 25% of its land being degraded. According to studies carried out across Ethiopia, natural vegetation, such as woods and scrublands, has suffered as cultivated areas have grown [8]. Studies carried out in Ethiopia by Bai et al. [9]; Belay et al. [10]; and Tesfa and Mekuriae [11] added that topography, unsustainable agriculture, fuelwood consumption, expansion of new agricultural land, a weak regulatory environment and institutions, unclear land use rights, low local community empowerment, poverty, and insufficient infrastructural development affect the dynamics of land use and land cover (LULC).

The study area was a place where agricultural surpluses were produced in 1971 [12], and today, it is one of the districts with a significant number of households suffering from food insecurity and supported by safety net programs [13]. In addition, the district was characterized by the gap between public-private partnerships in mitigating the effects of deforestation [14]; local conflicts between ethnic groups resulted from a competition for natural resources [15]. The district is also one of the places populated by an agricultural population with extensive farming activities; land areas, especially forests and shrub lands, have been under severe threat from time to time, and illegal logging and encroachment on protected areas still occur due to population growth and increasing demand for agricultural land [16].

Although several studies have been conducted on LULCC in Ethiopia, none of them have specifically analyzed the spatiotemporal dynamics of land use and land cover in relation to deriving forces and community perception in the Derashe district. Most studies focused on the watershed scale and failed to link land use/land cover change to causative forces and local community perceptions [17–24].

With these gaps in mind, this study aimed to quantify the land use and land cover dynamics from 1988 to 2019, as well as assess the deriving factors and local community perception towards land use and land cover change in the study area. The findings of this study provide valuable information for local administrative bodies and decision-makers.

## 2. Methods and Materials

### 2.1. Description of the Study Area

**Figure 1:**
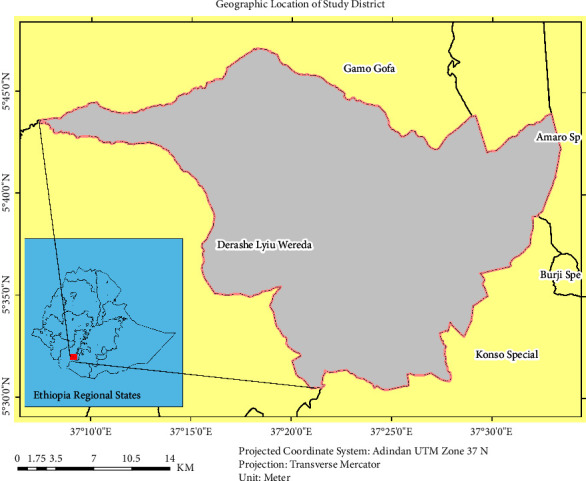
Geographic location of the study district.

Derashe district is one of the administrative districts in the SNNPRS in southern Ethiopia. It is located about 550 km from the capital, Addis Ababa, and 325 km from the city of Hawassa. It consists of 18 administrative kebeles, of which 16 are rural and two are urban [13]. Astronomically, the district extends from 5°30′00″ to 5°45′00″ N and 37°10′00″ to 37°35′00″ E (Figure 1). Its altitude (elevation) ranges from 501 m to 2500 m above the sea level [25]. Agroecologically, it is divided into three agroclimatic zones: Dega (highland or cool), Woyena Dega (midland or temperate), and Kola (lowland or hot) tropical. The long-term average maximum and minimum temperatures were 27.50°C and 15.10°C, respectively. Annual precipitation ranges from 601 mm to 1600 mm [26].

### 2.2. Methods

#### 2.2.1. Data Types and Sources

To achieve the objectives of the study, both primary and secondary data were used. Primary data were obtained from socioeconomic data from field studies, household surveys, KIIs, and FGDs. These data focused mainly on socioeconomic issues, living conditions, and local community perceptions of LULC's dynamic change, including the forces behind these dynamics. The impact of dynamic change and possible solutions were not ignored in the work. Secondary data used for the study include official reports, local and national CSA data, empirical studies, literature, ethio-GIS data, and land-satellite imagery (Table 1) from the USGS website (https://earthexplorer.usgs.gov/; retrieved December 24, 2019).

#### 2.2.2. Research Design and Method

A descriptive research method was developed to study the dynamics of land use and land cover as well as the perceptions of the local community in the study area. A purposive sampling technique was used to select districts, kebeles, members of KIIs, and members of FGDs, which was not a probability selection. The presence of adequate land use change, the absence of empirical studies on land use change in the study area, and the familiarity of the researcher were the reasons for selecting Derashe district as the study area. The three agroecological zones (Dega, Woynadega, and Kola) of the district were used as criteria for selecting three kebeles (Arguba, Walesa, and Holte) from the 18 kebeles in the district, while age (50 years), experience, and knowledge of the study area were the criteria for selecting members of the KII. In addition, gender, age (65), and educational background were used as criteria for selecting participants in the FGDs. As a result, 20 key informants (15 from selected kebeles and 5 at the district level) and 10 focus group discussions (9 at the kebele level and 1 at the district level) were conducted with six members each.

A descriptive research method was developed to study the dynamics of land use and land cover as well as the perceptions of the local community in the study area. A purposive sampling technique was used to select districts, kebeles, members of KIIs, and members of FGDs, which was not a probability selection. The presence of adequate land use change, the absence of empirical studies on land use change in the study area, and the familiarity of the researcher were the reasons for selecting Derashe district as the study area. The three agroecological zones (Dega, Woynadega, and Kola) of the district were used as criteria for selecting three kebeles (Arguba, Walesa, and Holte) from the 18 kebeles in the district, while age (>50 years), experience, and knowledge of the study area were the criteria for selecting members of the KII. In addition, gender, age (>65), and educational background were used as criteria for selecting participants in the FGDs. As a result, 20 key informants (15 from selected kebeles and 5 at the district level) and 10 focus group discussions (9 at the kebele level and 1 at the district level) were conducted with six members each. To determine sample size, the Kothari [27] formula was used.

(1) $$\begin{array}{c} n=\frac{z^{2}pqN}{e^{2}\left(N-1\right)}+z^{2}pq, \end{array}$$

where *n* is the sample size for a finite population, *N* is the size of population which is the number of households, *p* is the population reliability (or frequency estimated for a sample of size *n*), where *q* is 0.5, which is taken for all developing countries population and *p* + *q* = 1, *e* is the margin of error considered is 5% for this study *Z α*/2: normal reduced variable at the 0.05 level of significance z is a score value at the 95% confidence level. According to the above formula, the sample size for the study was

(2) $$\begin{array}{c} =\frac{{\left(1.96\right)}^{2}\;\left(0.5\right)\left(0.5\right)\left(5673\right)}{{\left(0.05\right)}^{2}\;\left(5673\right)}+0.96\\ =\frac{5448.3492}{15.1404}\\ =359.85\\ =360. \end{array}$$

#### 2.2.3. Analysis of the Dynamics of Land Use and Land Cover

To quantify LULC change dynamics, landsat imagery from the years 1988, 1998, 2010, and 2019 was downloaded from USGS websites. Prior to using these images as input data for classification, image preprocessing (layer stacking, radiometric correction, and topographic correction) was performed. Image enhancement is applied to the preprocessed data to effectively interpret the image for visual interpretation.

Image classification, the process of categorizing all pixels in an image to obtain a specific set of labels for land cover themes, was applied [28]. Supervised classification method was used to classify the images. Both ground control images and images from recent years were used in image classification, considering that they were acquired at comparable times during the study period [29]. In addition, Google satellites were used as the background for effective understanding and classification of the images. A maximum likelihood classification algorithm was used for the evaluation. The maximum likelihood classification algorithm is the most widely used, successful, and widely used classification algorithm [30]; Munthali et. al, 2013, as cited in [29]. Finally, a raster sieve was used to process misclassified pixels.

For this purpose, 50 stratified points were created for each LULC class in the classified image accounts and 350 points for each year using the semiautomatic classification plugin (scp). Then, the multiple points were converted into a shape file and checked against the historical images of Google Earth. Then, the multiple points were processed according to the LULC type they corresponded to in Google Earth, and the accuracy error matrix was calculated. For the reference years 1988, 1998, and 2010, for which the resolution of the historical Google Earth images caused some difficulties in identifying the correspondence of some points but not all, the researcher used the local prior knowledge, consistent coverage types that were not changed, and information from KIs to know what was found where, and classified images of recent years and false color interpretations of the image were used as the supporting material along with historical Google Earth images in the accuracy assessment. Finally, the error matrix was calculated by comparing the land use classifications of all the data with the available reference data.

The overall accuracy and Kappa statistics are calculated using the formula [31] as follows:

(3) $$\begin{array}{c} \text{over all accuracy}=\frac{\text{number of pixels correctly classified}}{\text{total number of pixels}}. \end{array}$$

Kappa (*K*^): It reflects the difference between actual agreement and the agreement expected by chance and is estimated as

(4) $$\begin{array}{c} K^{\wedge }=\frac{\text{Po}-\text{Pe}}{1-\text{Pe}}, \end{array}$$

where *Po* is the proportion of correctly classified pixels and determined by the diagonal in error matrix and *Pe* is the proportion of correctly classified pixels expected by chance and incorporates off-diagonal.

LULC change analysis was computed in three different ways: total LULC change in hectare is calculated as follows:

(5) $$\begin{array}{c} \text{total LULC change}=\text{area of final year}-\text{area of initial year}, \end{array}$$

where positive values suggest an increase and negative values imply a decrease in extent. Percentage change of LULC was calculated using the following equation:

(6) $$\begin{array}{c} \text{percentage change of LULC}=\frac{\text{area of }a\text{ final year}-\text{area of initial year}}{\text{area of initial year}}\times 100. \end{array}$$

Annual rate of LULC change is computed using the following simple formula.

(7) $$\begin{array}{c} r=\frac{Q2\;-Q1}{t}, \end{array}$$

where *r*, *Q*2, *Q*1, and *t* indicate the rate of change, recent year LULC in ha, initial year LULC in ha, and interval year between initial and recent years, respectively.

#### 2.2.4. Analysis of Socioeconomic Data

In this study, the main concern in integrating socioeconomic data with quantitative remote sensing data was to obtain additional information from the local community that would be helpful in explaining the results of the study in depth. Therefore, the socioeconomic data obtained from the household survey, KIIs, and FGDs were analyzed using the statistical package for social science software (SPSS version 20).

## 3. Results and Discussion

### 3.1. LULC Classes and Their Definitions

The major land use and land cover classification was defined according to the following description (Table 2).

### 3.2. Status of LULC Maps and Accuracy of the Classification

**Figure 2:**
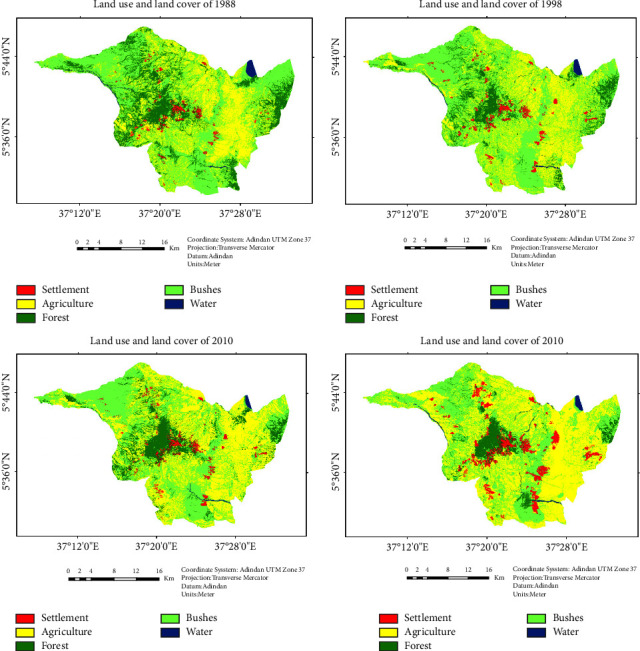
Land use land cover maps of the study area.

In Figure 2, four LULC maps are created with five categories of LULC classes. These are settlement areas, agricultural areas, forest areas, scrublands, and water bodies. The overall accuracy of the 1988, 1998, 2010, and 2019 maps was 92.05%, 89.25%, 89.96%, and 95.85%, respectively, with a kappa coefficient of 86.86%, 81.66%, 84.11%, and 93.01%, respectively.

Table 3 shows the land use and land cover classes in hectors for 1988, 1998, 2010, and 2019. Settlements had the lowest land cover in 1988 at 1.50%, which increased to 4.90% in 2019. Agriculture had the highest area coverage in all years, starting at 29.30% in 1988 and increasing to 50.15% in 2019. Forest and bush had fluctuating area percentages over the years. The forest had a peak of 16.97% in 1988, decreasing to 6.59% in 2019. On the other hand, the bush had a peak of 54.00% in 1998, which decreased to 37.61% in 2019. Finally, water had the lowest area coverage in all years, ranging from 0.54% to 0.85%.

### 3.3. LULC Dynamics within the Referenced Periods 1988–2019

Settlements increased across all time periods at rates of 49.338, 24.89, 176.42, and 76.77 hectares per year from the starting year (1988) to the ending year (2019) (Table 4). This is mainly related to the resettlement in 1989 and the strong population growth that was addressed by the FGD participants. As a result, the settlement area in the study area continued to increase.

Agricultural land has changed dramatically, with an increased rate of 261.3–619.02 ha/year between 1988 and 2019 (Table 4). Agricultural land was the largest land cover of the study area with a size of 71.12% at the end of the referenced year 2019 (Table 4), which can be attributed to the combination of economic and sociocultural underlying deriving forces.

While the forest land decreased in the three reference years, except in 2010, when it increased by 13.39%, this was due to the focus on natural resource management in the study area. However, in general, it decreased from 16.97% in the baseline year of 1988 to 6.59% at the end of the reference year of 2019 (Table 4).

Next to settlements, water bodies were the lowest LULC class, covering 0.54% to 0.86% of the landscape between 1988 and 2019 (Table 4). The change in the LULC class of water bodies continued at a decreasing rate of 7.12–12.46 ha/year in the first two periods (1988–1998 and 1998–2010) and increased by 36.08 ha in 2010–2019 (Table 4).

### 3.4. LULC Conversion Matrix

The conversion matrix was calculated to show the gain and loss of LULC change in Derashe district.  Table 5 clearly illustrates the source of the increase and the target of the loss of land cover. During the study periods 1988–1998, 1998–2010, 2010–2019, and 1988–2019, 40.16%, 37.24%, 39.94%, and 50.51% of the total landscape in the study area were converted from one LULC type to another, respectively. During 1988–1998, most of the settlement (71.91 ha) and water (111.6 ha) areas were converted to bush land. During the same period, 8142.39 ha of agricultural land was converted mainly to bush land, with most of the forest and bush land converted to agricultural land.

Between 1998 and 2010, the main settlement land (86.67 ha) was converted to forest land, followed by 64.53 ha converted to agricultural land, while agricultural, forest, and water areas were converted to bush land. In turn, most of the bush land was converted to agricultural land. Also, in 2010–2019, most of the settlement land was converted into agricultural land, while agricultural, forest, and water land were converted into bush land. However, most of the bush land was converted into agricultural land (Table 5). On the other hand, in the last 31 years between 1988 and 2019, 1.5 to 4 hectares per year of water bodies were converted into agricultural land.

At the end of the reference year between 1988 and 2019, 49.5% of the landscape of the study area remained unchanged, while 50.5% was converted from one land cover to another. As a result, settlement and agricultural lands had a net gain of 2379.87 ha and 14577.75 ha, respectively, while forest lands, bushlands, and water bodies lost 7261.29 ha, 9611.64 ha, and 84.69 ha, respectively, at the end of the reference year (2019).

### 3.5. Perception on the Deriving Forces of LULC Dynamics

#### 3.5.1. Perceptions on Proximate Deriving Forces

The majority of respondents blamed agricultural expansion for the LULC dynamics in the district. Demand for natural resources was the second responsible deriving force in Derashe district (Table 6). In terms of natural resources demanded, demand for firewood for domestic use ranked first, followed by demand for construction and timber among the demanded natural resources. However, demand for charcoal for domestic use and sale played no role in LULC changes (Table 6). Settlement expansion was ranked third. The majority of respondents indicated that large numbers of livestock (overgrazing) were not a proximate deriving force of LULC change in Derashe District.

#### 3.5.2. Perception on the Underlying Deriving Forces of LULC Dynamics in Derashe District

Rapidly growing population pressure is one of the major deriving forces of LULC changes that put pressure on limited land for agricultural production. Accordingly, the majority (64.9%) of the surveyed households indicated that demographic trends were the most important drivers of land use and land cover changes in the study area. Economic, institutional, and sociocultural factors, on the other hand, ranked second through fourth (Table 7). As outlined in most KIIs and FGDs, population growth forced farmers to cultivate any land they could get, including steeper slopes and closed areas. This type of farming in turn exacerbates the high surface runoff that displaces them from their farmland.

The attachment of community to the land for economic source rather than nonagricultural source of income was among the predominant underlying deriving force for the dynamics of LULC that has been aggravating the expansion of agricultural land. Furthermore, one of the KIIs indicated that owing to the economic deriving force, even cultural beliefs have been disrupted and the cultivation of sepulcher (*Meqabir*) areas has started.

Institutional and sociocultural underlying deriving forces were ranked third and fourth by the majority of the respondents. An undermined number (45–65%) of respondents perceived that attitude, beliefs, and collective memories of the community contributed to LULC changes in the study area. In addition, 35–69% of the respondents perceived that the weak regulation, land tuner system, and unclear user rights were the institutional deriving forces in the study area. Most of the KIIs and FGDs stressed up on the linkage of institutional and sociocultural underlying deriving forces. The existing land tuner system, which is a cultural land holding system, leads to unlimited illegal logging, and the behavior of the regimes of the country was identified as institutional deriving forces in the study area. Additionally, attitudes of the community and cultural beliefs such as the absence of honor and respect for nonagricultural business activities were among the identified and presented underlying deriving forces of LULC changes.

#### 3.5.3. Perception on the Effect of LULC Change

The majority of the respondents (81.4%–96.4%) realized that there had been LULC changes of the effect on both individual households and community as a whole. Among the identified effects, the effects of floods, deforestation, loss of crop production, reduction of grazing land, loss of soil fertility, and loss of animal production were identified as severe problems by 69.7%, 55.8%, 55.6%, 52.5%, 60.3%, and 40.3% of the household respondents, respectively. On the other hand, siltation and sedimentation, food shortage, reduction of bio-diversity, displacement from crop land and settlements, and death of livestock were confirmed by 30% to 37.8% of the respondents as a severe effect in the study area.

### 3.6. Perception on the Remedies Taken Place and Indicated Potential Solutions

Based on the reasons for the failure of the remedies, the KIIs and FGDs indicated the following potential solutions:

1. Shifting the community livelihood to nonagricultural income sources that reduce their livelihood dependence on farmland and improve their welfare
2. Conducting continuous capacity building activities until the community decides to shift their mind to nonagricultural business activities as a solution for the effect of LULC change rather than illegal logging into hill sides and marginal areas
3. Develop a project in which the community gets potential income from forest and bush lands without destroying them, for example, honey and spices production
4. Trust building among the elites, political leaders, and the community for positive flow of ideas during community mobilization and capacity building trainings

### 3.7. Discussion

The changes in LULC indicated a significant shift towards settlement and agricultural expansion, while forested areas have experienced a notable decline. Bush land areas have also decreased over the studied period. The rates of change vary for each category, with settlement and agricultural areas showing higher growth rates compared to the decline rates observed in forests and built-up areas. The percentage change and rate of LULCC in four different categories were presented as follows.

In the year interval 1998–2010, a small increase (298 ha) and rate (25 hectares per year) of change were identified. From 2010 to 2019, the substantial increase in settlement area at 1587 hectares (86.2% change) with a growth rate of 176 hectares per year was observed. It looks like that the late 1980s' resettlement was contributed to this change. Over the past 31 years (1988 to 2019), settlements were expanded by 2380 hectares (227% change) with an average annual growth rate of 77 hectares. This is consistent with the findings of Kindu et al. [32] and Agidew and Singh's [33] studies of Munessa-Shashemene and Teleyayen subwatersheds in the northeastern highlands of Ethiopia.

Agricultural land increased significantly (12.74%) between 1988 and 1998, with a growth rate of 261 hectares per year. Subsequently, from 1998 to 2010, agricultural land doubled with an increase of 6393 hectares (28%) and a growth rate of 533 hectares per year. The increased demand for agricultural land due to population growth in the study area contributed to the observed change. This is consistent with the study conducted by Megersa [34]. Between 2010 and 2019, the increase in agricultural land was slowed. In 2007, the regional government promulgated the Proclamation on the Management and Use of Land [35]. It states that rural land that is sloppy and degraded must be protected from human and animal contact. This hindered the expansion of agricultural land. Similarly, in the Dedza region of central Malawi, agricultural land area decreased from 71.3% to 69.41% at the end of the reference year [29], and in the study in the Wujig-Mahgo Waren Forest in northern Ethiopia, Kiliso subwatershed of Oromia region, an agricultural land area, declined considerably [36, 37].

Between 1988 and 1999, there was a notable reduction in both the area and rate of change in forests. This downward trend continued at a slower pace from 1998 to 2010, with fluctuations over the past 31 years. On average, the forest area decreased by 7261 hectares (−61%) between 1988 and 2019, with an annual decline rate of 234 hectares. Similar findings were observed in a study conducted in central, southern, and northern Ethiopia [36–38], where the forest area increased during one period and decreased during another due to changes in forest land management. This is different from the studies conducted in Ethiopia where a decrease in forest area was found in all study periods [32, 33, 39, 40] and [34].

The LULC change transition matrix provides valuable insights in to the changes that have occurred in different LULC classes overtime. It reveals a significant conversion between different LULC classes. The resettlement that took place during the Dergu regime from late 1988 to 1989 within the district was raised in most KIIs and FGDs as a deriving force that caused the conversion of agricultural land and bush land to settlements and in turn the conversion of settlements to bush and forest land. This suggests that this study is consistent with the study conducted in the Abaya and Chammo basins [41] in confirming resettlement as a driver of LULC dynamics. The largest share of water bodies (188.91 ha) was converted into bush land over the last 31 years. This is due to the decline and increase of Lake Chamo and the expansion of agricultural land on the side land where the lake dried up. This was practically confirmed in the field and in the historical images on Google Earth. This is due to the fluctuation of Lake Chamo, which shrank by 9.3% between 1985 and 2010 and increased after 2010 due to Lake Abaya runoff as a form of overflow and increased surface runoff from the watershed [41].

The majority of the local community perceives that agricultural expansion is the major proximate cause of LULC dynamics in succeeding demand for natural resources. This perception of the surveyed households matched well with the findings from the image classification analysis. This is consistent with the study conducted by Betru et al. [42] and Tadesse et al. [43], which identified agricultural expansion as the main driver of LULC change. As Gessesse noted, demand for wood for construction materials contributed to uncontrolled land cover change and deforestation in Ethiopia [1]. The majority of the respondents were not considered overgrazing as a proximate factor. This was contrary to the studies that accepted overgrazing as a proximate deriving force [1, 5, 34]. This study confirmed the FAO idea that there are significant regional differences in the drivers of LULC change [4].

The majority of the surveyed households indicated that demographic trends were the most important underlying drivers of land use and land cover changes in the study area. This is consistent with different researchers finding that as the human population increased, the demand for farmland was inevitable [34, 44, 45]. The major identified effects of land use/cover change include flooding, deforestation, loss of crop production, reduction of grazing land, loss of soil fertility, and loss of animal production. On the other hand, siltation and sedimentation, food shortage, reduction of bio-diversity, displacement from crop land and settlements, and death of livestock were confirmed as a severe effect in the study area.

## 4. Conclusion

The changes in land use and land cover indicate a significant shift towards settlement and agricultural expansion, while forested areas have experienced a notable decline. Bush land areas have also decreased over the studied period. The rates of change vary for each category, with settlement and agricultural areas showing higher growth rates compared to the decline rates observed in forests and built-up areas. The major proximate deriving force was the agricultural expansion followed by demand for natural resources, natural forces, and settlement expansion ranked from second to fourth by the majority of the respondents, while a large number of animals on grazing land and mining were perceived as invisible proximate deriving forces in the study area. On the other hand, the predominant underlying deriving forces are socioeconomic characteristics of the local community (demographic and economic forces) which in turn leads to increasing demand for land and forest products.

The observed LULC changes have had severe negative implications on the livelihoods of local communities. The steady decline of forest land will trigger soil erosion and sedimentation. This could exacerbate land degradation, loss of agricultural land, and failurity of crop production. The study area is already experiencing flooding, deforestation, loss of crop production, reduction of grazing land, loss of soil fertility, and loss of animal production. The remedies implemented to minimize the effects of LULC change have not been successful without changing the socioeconomic base of the local community from agricultural to nonagricultural businesses. This is due to the seasonal nature of the agriculture-based economic income source and the sociocultural attitudes of the local community. It is a critical solution to save the community from the vicious circle of LULC change effects that result from wrong measures taken to cope with the effects of LULC change (such as illegal logging in forests and reserved areas) and to attain the sustainable use of natural resources. Therefore, to mitigate the unfavorable LULC change impacts and improve the livelihood of the local community, it is vital to shift the agricultural-based income source of the community to nonagricultural business activities, in addition to strengthening the ongoing remedies.

## Figures and Tables

**Table 1:** Landsat images used in the study.

Satellite image	Acquisition year	Sensor (identifier)	Path/row	Spatial resolution (m)	Cloud cover	UTM zone	Datum	Map projection	Source
Landsat 4-5	03/12/1988	TM	169/56	30^*∗*^30	0	37	WGS-84	UTM	USGS
Landsat 4-5	31/12/1998	TM	169/56	30^*∗*^30	0	37	WGS-84	UTM	USGS
Landsat 4-5	30/01/2010	TM	169/56	30^*∗*^30	0	37	WGS-84	UTM	USGS
Landsat 8	23/01/2019	OLI-TIRS	169/56	30^*∗*^30	0.43	37	WGS-84	UTM	USGS

**Table 2:** Description of LULC classes, field survey 2022.

No.	LULC class	Description	Photo
1	Settlement	Residential (urban & rural), socioeconomic infrastructure (transportation (roads, stations), tel communication, and utilities), and mixed residential lands	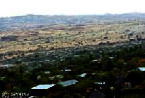

2	Agricultural	All cultivated and uncultivated agricultural lands such as crop fields, orchards, groves, vineyards, nurseries, and ornamental horticultural areas, farmsteads, corrals, small farm ponds, ditches, and canals in the farm	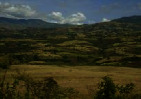

3	Forest	Natural forests, plantation forest (man-made), mixed forest lands and forests on customary land	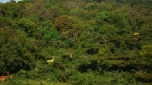

4	Bush	Land covered by scattered various woody shrubs and bushy small trees occur in dense-to- open thickets and mixed with grass vegetation including bare land; wet land and grass/range lands found mostly in hilly areas and closures water shades	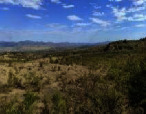

5	Water body	Rivers (including dry rivers visible on the image) and permanent open water bodies	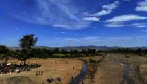

**Table 3:** Absolute area coverage of the LULC classes; classification result, 2022.

No.	LULC class	Absolute area cover of the classes in hector over the periods							
		1988		1998		2010		2019	
		Area	%	Area	%	Area	%	Area	%
1	Settlement	1049.58	1.50	1542.96	2.21	1841.67	2.63	3429.45	4.90
2	Agriculture	20497.59	29.30	23110.83	33.05	29504.16	42.19	35075.34	50.15
3	Forest	11874.24	16.97	6987.42	9.99	7923.6	11.33	4612.95	6.59
4	Bush	35917.47	51.35	37768.86	54.00	30290.22	43.31	26305.83	37.61
5	Water	597.96	0.85	526.77	0.75	377.19	0.54	513.27	0.73

	Total	69936.84	100	69936.84	100	69936.84	100	69936.84	100

**Table 4:** LULC change percentage and annual rate in the referenced periods; classification result 2022.

Percentage change (ha) in each period of time (ha)												
LU class	1988–1998			1998–2010			2010–2019			1988–2019		
	ha	%	rat	ha	%	rat	ha	%	rat	ha	%	rat
Set	493	47.01	49	298	19	25	1587	86.2	176	2380	227	77
Agr	2613	12.74	261	6393	28	533	5571	18.8	619	14578	71	470
Fo	4887	−41.15	−489	936	13	78	−3310	−41.7	−368	−7261	−61	−234
Bu	1851	5.15	185	−7478	−2	−623	−3984	−13.1	−443	−9611	−27	−310
Wa	−71	−11.9	−7	−149	−28	−12	136	36	15	−84	−14	−3

NB, Set: settlement, Agr: agriculture, Fo: forest, Bu: bush, Wa: water, and rat: rate.

**Table 5:** Conversion matrix of land class classification result 2022.

No.	From	To	1988–1998	1998–2010	2010–2019	1988–2019
1	Settlement	Agriculture	17.28	64.53	172.98	99.72
		Forest	8.55	86.67	149.85	112.59
		Bush	71.91	58.41	146.52	88.56
		Water	0	0	0.45	0.09

2	Agriculture	Settlement	223.02	156.15	764.19	909.9
		Forest	347.49	507.87	154.53	224.37
		Bush	8142.39	6572.16	7514.37	5183.55
		Water	20.97	17.1	37.71	35.46

3	Forest	Settlement	71.73	71.73	325.08	355.77
		Agriculture	1299.33	43.02	1259.1	3806.64
		Bush	5613.3	1992.96	2813.49	4581.99
		Water	7.65	3.24	24.84	19.98

4	Bush	Settlement	296.19	309.15	967.86	1414.98
		Agriculture	10004.76	12430.08	12594.87	16962.75
		Forest	1710.36	3436.92	806.04	1165.23
		Water	76.5	63.72	138.69	111.69

5	Water	Settlement	0.18	0	0.45	0.18
		Agriculture	25.74	43.29	15.03	61.92
		Forest	38.79	52.65	1.44	0.9
		Bush	111.6	137.7	48.69	188.91

**Table 6:** Rank of proximate deriving forces, field survey 2022.

Types of proximate deriving forces		First	Second	Third	Fourth	Fifth
Demand for natural resources	Count	101	142	86	20	0
	%	28.9%	40.7%	24.6%	5.7%	0.0%

Agricultural expansion	Count	218	98	31	2	0
	%	62.5%	28.1%	8.9%	0.6%	0.0%

Large number of animals on grazing	Count	0	0	5	10	19
	%	0.0%	0.0%	14.7%	29.4%	55.9%

Settlement expansion	Count	22	102	150	55	4
	%	6.6%	30.6%	45.0%	16.5%	1.2%

Mining	Count	0	0	4	17	47
	%	0.0%	0.0%	5.9%	25.0%	69.1%

Natural force	Count	13	20	77	207	7
	%	4.0%	6.2%	23.8%	63.9%	2.2%

**Table 7:** Rank of major underlying deriving forces, field survey 2022.

		First	Second	Third	Fourth	Fifth	Sixth
Economic forces	Count	105	176	44	15	2	2
	%	30.5%	51.2%	12.8%	4.4%	0.6%	0.6%

Demographic forces	Count	222	100	17	1	2	0
	%	64.9%	29.2%	5.0%	0.3%	0.6%	0.0%

Sociocultural forces	Count	16	42	104	94	37	9
	%	5.3%	13.9%	34.4%	31.1%	12.3%	3.0%

Institutional forces	Count	11	27	125	97	33	8
	%	3.7%	9.0%	41.5%	32.2%	11.0%	2.7%

Biophysical forces	Count	3	15	46	101	142	20
	%	0.9%	4.6%	14.1%	30.9%	43.4%	6.1%

Technological forces	Count	1	3	12	29	55	128
	%	0.4%	1.3%	5.3%	12.7%	24.1%	56.1%

## Data Availability

The data that support the findings of this study are included within the article.
